# A Convolutional Neural Network to Identify Parkinson’s Disease using Multimodal Retinal Images

**DOI:** 10.1002/alz.089793

**Published:** 2025-01-09

**Authors:** Alexander Richardson, Anita Kundu, Ricardo Henao, Terry Lee, Burton L. Scott, Dilraj S Grewal, Sharon Fekrat

**Affiliations:** ^1^ Duke University, Durham, NC USA; ^2^ Duke Eye Center, Durham, NC USA; ^3^ Duke University School of Medicine, Durham, NC USA; ^4^ Duke Department of Neurology, Durham, NC USA

## Abstract

**Background:**

Retinal structure and microvasculature may be used as a surrogate for parallel processes in the brain. Previous studies have revealed differences in retinal structure and microvasculature in patients with Parkinson’s disease (PD) compared to cognitively normal controls[1]. Previous work developed a convolutional neural network (CNN) trained on multimodal retinal images that was able to identify Alzheimer’s dementia with an area under the curve (AUC) of 0.841[2]. Herein, we developed a CNN to identify PD.

**Methods:**

Building upon a pretrained VGG19 architecture, we trained a CNN to receive inputs of ganglion cell‐inner plexiform layer (GC‐IPL) thickness color maps, optical coherence tomography angiography (OCTA) superficial capillary plexus 6x6mm en face images centered on the fovea obtained using the Zeiss Cirrus HD‐5000 with AngioPlex (v11.0.0.29946), ultra‐widefield (UWF) fundus color and autofluorescence (FAF) scanning laser ophthalmoscopy (Optos California) images from patients with a clinical diagnosis of PD and from controls without PD or cognitive impairment. The model consisted of a shared convolutional encoder, parallel image modality specific feature transformations, and prediction heads for all modalities that converge into a single output.

**Results:**

1,784 images from 371 eyes of 249 control subjects and 75 eyes of 52 PD subjects were used for development and testing. 296 (80%) control eyes were used for training, 36 (10%) for validation, and 39 (10%) for testing. 59 (79%) PD eyes were used for training, 6 for validation (8%), and 10 (14%) for testing. Our best performing model had an AUC of 0.918. UWF color images proved to be the most effective imaging input (highest AUC 0.838), and GCL‐IPL color maps were the least effective (highest AUC 0.630).

**Conclusions:**

This CNN was able to successfully identify individuals with a clinical diagnosis of PD. Further work is ongoing to expand the number of images and the diversity of the population to improve translatability.

Ma, J.P., et al., Retinal vascular changes in Parkinson’s disease on ultra‐widefield retinal imaging. Investigative Ophthalmology & Visual Science, 2021. 62(8): p. 1779‐1779.

Wisely, C.E., et al., Convolutional neural network to identify symptomatic Alzheimer’s disease using multimodal retinal imaging. British Journal of Ophthalmology, 2020. 106: p. 388 ‐ 395.

